# 3M Syndrome:  A Report of Four Cases in Two Families

**DOI:** 10.4274/jcrpe.v3i3.30

**Published:** 2011-08-09

**Authors:** Ayla Güven, Ayşe Nurcan Cebeci

**Affiliations:** 1 Göztepe Educational and Research Hospital, Pediatric Endocrinology Clinic, Istanbul, Turkey; +90 532 238 03 00 aylaguven@yahoo.com

**Keywords:** 3M syndrome, short stature, slender bone

## Abstract

3M syndrome is a rare entity characterized by severe growth  retardation, dysmorphic features and skeletal changes as its major  components. It is differentiated from other types of dwarfism by its  clinical features and by the typical slender long bones and

foreshortened vertebral bodies that can be visualized radiographically. 3M syndrome has an autosomal recessive mode of inheritance. An early diagnosis is important for genetic counseling. In this report, we present four children (3 males, 1 female) from two families who were aged between 411/12 and 1011/12 years and had clinical findings of 3M  syndrome. One of these patients had received growth hormone (GH) treatment which was discontinued due to an inadequate height gain. Physicians should be aware of this entity in the differential diagnosis of children with severe short stature and mild skeletal changes.

**Conflict of interest:**None declared.

## INTRODUCTION

3M syndrome (OMIM #273750) is characterized by severe dwarfism, low birth weight and dysmorphic features. It was first described by Miller, McKusick and Malvaux in 1975 (1). It is rare a syndrome with unknown prevalence.  The diagnosis of 3M syndrome is based on clinical and radiological findings. Intrauterine growth retardation, severe short stature and  skeletal changes are the main characteristics of this syndrome.

  The aim of this paper is to add four new patients from two different families to the literature on this syndrome.  

## CASE REPORTS

**Family 1 (Patients 1 and 2) Patient 1**

**Figures 1A:**
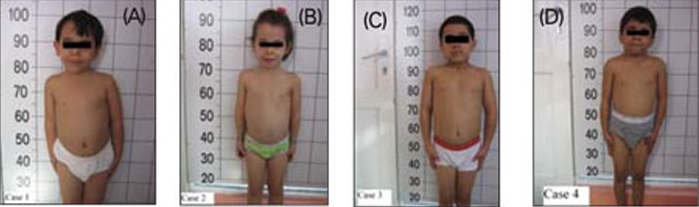
Figure 1. Patients 1 (A), 2 (B), 3 (C) and  4 (D). The patients exhibited the physical findings of  3M syndrome: Dolichocephaly, high and broadforehead, upswept posterior hairline (in patients 1 and 2), downward slanting of the eyes, horizontal eyebrows, flat nasal bridge, narrowed and short nasal body, prominent fleshy nasal tip, straight clavicles, high and square shoulders, short, wide and flat thorax with a mild pectus excavatum

A 62/12-year-old boy was referred to our endocrine  clinic for marked short stature. Following an uncomplicated pregnancy, the patient was born at term by Caesarean section because of breech presentation (Table 1). His anthropometric measurements at birth and at admission are given in Table 2. The patient was the first child of healthy first-cousin parents. His motor and mental development were normal. Physical examination findings were compatible with 3M syndrome and are presented in Table 1 and in Figures 1A and 2A. 

 Complete blood count and biochemical analysis were  unremarkable. Thyroid function tests were in the normal range. The patient was negative for anti-endomysial antibodies (EMA) IgA. He had a normal response to growth hormone (GH)  stimulation test with clonidine (peak GH level was 19.6 ng/mL). Physiological GH profile during sleep was within normal limits (mean GH: 7.6 ng/mL). Insulin-like growth factor-1 (IGF-1) 

generation test for excluding GH insensitivity syndrome (GHIS) was normal (Table 3). Due to phenotypic findings in this boy and his sister (Case 2), the family was referred for genetic counseling. Radiographic bone survey showed slender long bones with  diaphyseal constriction and flared metaphyses, slender ribs, thick cortex of the tibia and femur, tall lumbar vertebrae, a small pelvis, short femoral neck and short iliac wings ([tif:Figure 3. Lateral radiography of the vertebrae showing tall vertebral bodies with reduced anterior-posterior diameters (Patient 1)|9-3.tif]Figures 3[/tif] and [tif:Figure 4. Radiography of the upper and lower extremities showing slender long bones (Patient 1)|9-4.tif]4[/tif]). Abdominal ultrasonographic examination was normal. 

**Patient 2**

**Figure 5:**
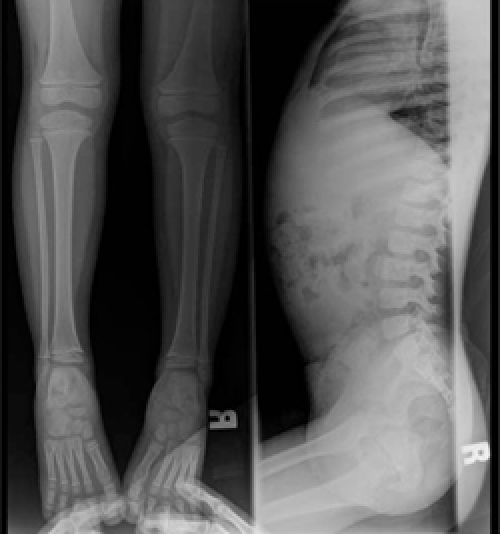
Figure 5. Radiography of the lower extremity and lateral vertebrae in Patient 2

This patient was the younger sister of Patient  1. She was born at term after an uncomplicated pregnancy and Caesarean section because of breech presentation (Table1). Her birth weight and length are given in Table 2. Her developmental milestones were achieved within normal time frames.  Anthropometric measurements at  age 411/12 years are given in Table 2. The findings in her physical examination were very similar to those of her brother (Figure 1B). Results of complete blood count and biochemical analysis were unremarkable. Thyroid function tests were in the normal range. EMA IgA was negative.   The patient had a normal response to GH stimulation  test with clonidine (peak GH level was 40 ng/mL). GHIS was  excluded with IGF-1 generation test (Table 3). Skeletal  survey showed slender long bones with diaphyseal  constriction and flared metaphyses, slender ribs, tall lumbar vertebrae, small pelvis and short iliac wings (Figure 5). Abdominal ultrasonographic examination was normal. The phenotypic findings in these sibs in combination with skeletal radiography and consanguinity in the family led us to a diagnosis of 3M syndrome. Unfortunately the parents refused molecular genetic analysis. 

**Family 2 (Patients 3 and 4)**

**Patient 3**

**Figure 7:**
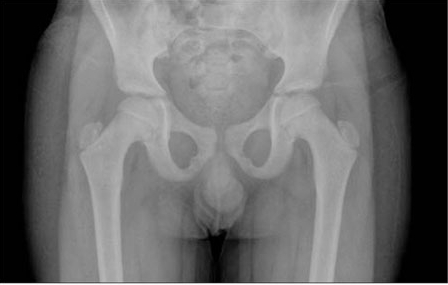
Figure 7. Note the narrow pelvis, hypoplastic pubis and ilium, as well as the short femoral necks (Patient  3)

This 1011/12 -year-old boy was referred to our clinic for severe short stature. He was the first child of first-cousin  parents. He was born small for gestational age (Table 1). His developmental milestones were achieved within normal  limits. He had incomplete response to GH stimulation as well as to exogenous GH therapy given for two years in another pediatric endocrinology department and the treatment had been stopped. His physical and skeletal findings were not compatible with GH deficiency. The skeletal dysplasia was not considered at genetic evaluation at age 9 and GH  therapy was recommended.  GHIS was excluded with IGF-1 generation test (Table 3).   The patient’s anthropometric and physical examination findings are given in Table 1 and in Figures 1C, 2B and 6. His biochemical and hormonal results were within normal limits. Skeletal survey of the patient showed slender long bones with diaphyseal constriction and flared metaphyses, slender ribs, thick cortex in the tibia and femur, short femoral neck, tall lumbar vertebrae, small pelvis and short iliac wings (Figure 7). 

**Patient 4**

**Figure 8:**
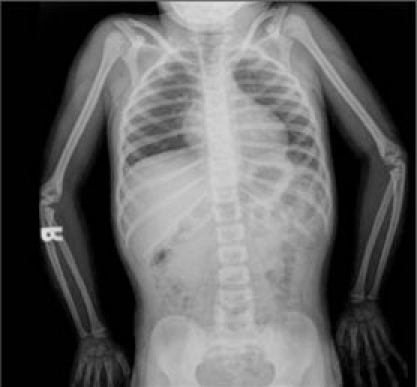
Figure 1. Patients 1 (A), 2 (B), 3 (C) and  4 (D). The patients exhibited the physical findings of  3M syndrome: Dolichocephaly, high and broadforehead, upswept posterior hairline (in patients 1 and 2), downward slanting of the eyes, horizontal eyebrows, flat nasal bridge, narrowed and short nasal body, prominent fleshy nasal tip, straight clavicles, high and square shoulders, short, wide and flat thorax with a mild pectus excavatum

This boy was the younger brother of Patient 3 (Figure 1D).  At admission, the patient was 74/12 years old. He suffered from short stature, as his brother.  His weight and length at birth and at admission are given in Tables 1 and 2. His developmental  milestones were achieved within normal limits. The boy had testicles small for his age. He had a normal response to GH stimulation test with clonidine (peak GH level was 18.2 ng/mL). GHIS was excluded with IGF-1 generation test (Table 3).  Skeletal survey showed slender long bones with diaphyseal constriction and flared metaphyses, slender ribs, hypoplasia of the 12th ribs, tall lumbar vertebrae, short femoral neck, a small pelvis, and short iliac wings (Figure 8). Abdominal  ultrasonography was normal. The diagnosis of 3M syndrome was established. Genetic analysis could not be performed because the family moved to a different city.  

**2A:**
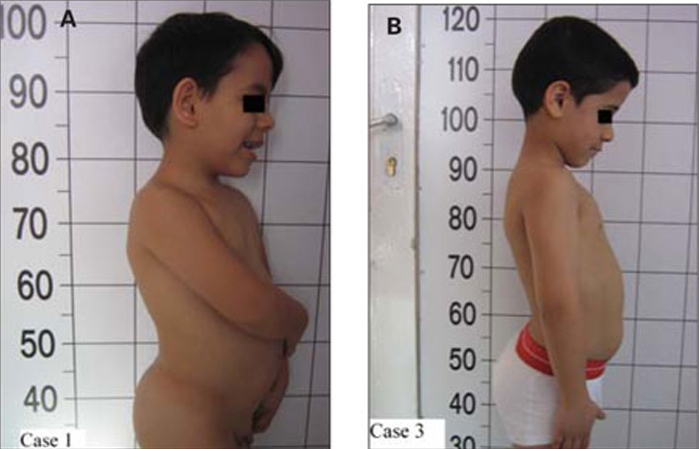
Figure 2. Exaggerated hyperlordosis as seen in Patients  1 (A) and  3(B)

**6:**
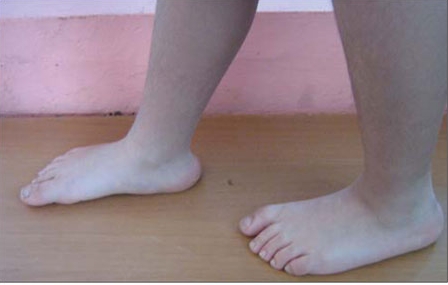
Figure 6. Prominent heels were present  in all patients

**Table 1:**
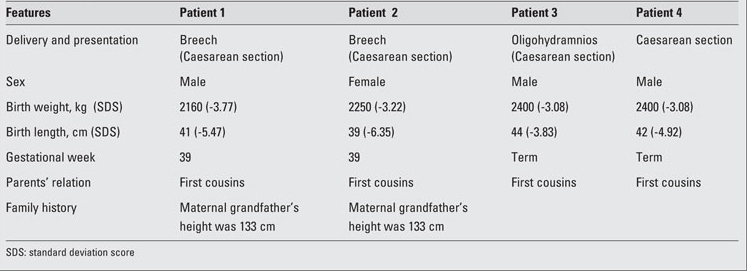
Table 1. Data on patient histories

**Table 2:**
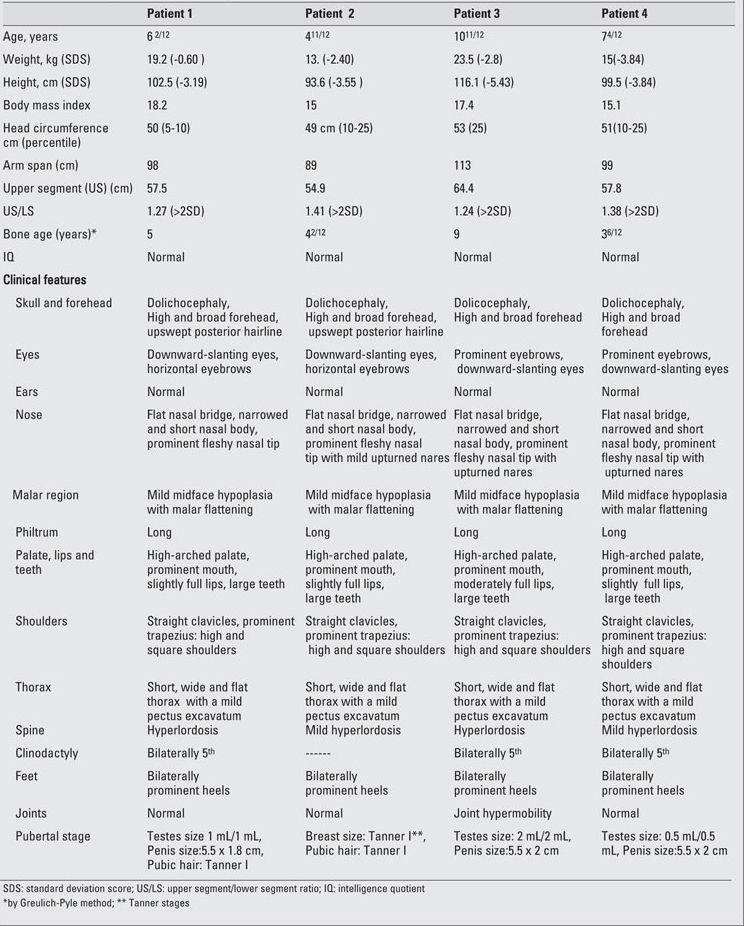
Table 2. Clinical findings of the patients (at diagnosis)

**Table 3:**
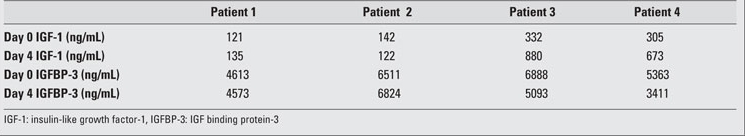
Table 3. Results of the IGF-1 generation tests

## DISCUSSION

Four patients from two different families who all suffered from severe short stature are presented in this report. All patients were also born small for gestational age. The clinical, radiographic and biochemical findings of all 4 patients  conformed the findings characteristic for 3M syndrome. 

 3M syndrome is characterized by a triangular-shaped face with frontal bossing, mild malar hypoplasia, and narrowed nasal body with a fleshy tip, upturned nares and full lips. Patients  usually have large heads for their height, dolichocephaly and  normal intelligence. Other clinical findings are a short wide  thorax, brachydactyly, clinodactyly, micromelia, prominent heels and a slightly increased upper to lower segment ratio. Both sexes are affected equally. No hormonal deficiencies are  detected. Skeletal changes include tall vertebral bodies and  cylindrical long bones with thin diaphyses. The radiographic examination, though abnormal, is not diagnostic as similar X-ray changes have been documented in other disorders (2,3,4). The patients with 3M syndrome cannot be easily  diagnosed due to slightly dysmorphic facial features and  normal intelligence. All four cases were diagnosed after  multiple visits and follow-up by different disciplines including genetic evaluation. 

 Presence of GH deficiency is usually evaluated in patients with 3M syndrome. Miller et al (1) have reported one patient with partial GH deficiency. All patients presented here  underwent GH stimulation tests and normal GH responses were obtained in all except Patient 3. This boy was treated with GH for two years, however, an insufficient growth response was observed and the therapy was discontinued. IGF-1 generation test was also performed to exclude growth hormone insensitivity. The GHIS is characterized by  hypoglycemic episodes, severe growth failure, and a typical craniofacial appearance. Biochemically, GH levels are elevated combined with extremely low levels of IGF-1, IGF-binding  protein-3 (IGFBP-3), and IGF-2. GHIS is confirmed by the  failure of exogenously administered GH to elevate the levels of IGF-1 or IGFBP-3 significantly. Although no hypoglycemic episodes and typical craniofacial appearance were present in any of our patients, they all had severe growth failure;  therefore, they underwent IGF-1 generation tests. 

 Gloomy face syndrome and dolichospondylic dysplasia formerly were reported as entities different from 3M  syndrome (5,6). Nowadays, the clinical and radiographic  findings of both disorders are found to overlap with 3M  syndrome and, 3M syndrome, gloomy face syndrome and dolichospondylic dysplasia have the same OMIM identification number (5,6). 

 Physical findings of several entities such as Silver-Russell syndrome (SRS) and Mulibrey nanism are similar to 3M  syndrome. SRS has many similarities with 3M syndrome, e.g. intrauterine growth retardation, short stature, triangular face, relatively large skull, asymmetry of body or limbs and   clinodactyly. Mild mental retardation also can be found in patients with SRS. However, abnormalities of the skeletal  system have not been reported.  Some cases with SRS have maternal disomy for chromosome 7. Chromosomes 11 and 17 are also consistently involved in individuals fulfilling strict  diagnostic criteria of SRS (7).  Recurrence in a sibship is much less frequent. Adult height of patients with SRS can be as tall as 140 cm, while patients with 3M syndrome do not reach a height greater than 130 cm. 

Mulibrey nanism (muscle-liver-brain-eye) is an autosomal recessive disorder characterized by prenatal and postnatal growth retardation, relatively large hands, and triangular facies with frontal bossing. Depressed nasal bridge, elongated sella turcica, and cystic bone changes of the tibiae are also  common findings of the syndrome (8).

  Maksimova et al (9) have reported 43 patients with short stature, hydrocephaloid skull and typical face in an isolated Yakut population. Although clinical findings were similar to 3M syndrome, slender long bones and tall vertebral bodies have not been commonly observed in this short stature syndrome in Yakuts (9).

 Bone age, estimated by the Greulich and Pyle method, was found to be markedly decreased in only one of our male patients. Delayed bone age (2,10) as well as bone age  concordant with chronological age (3) have been reported in  3M syndrome patients. Testicles small for pubertal stage, oligo-azoospermia and hypergonadotropic hypogonadism have been reported in males with 3M syndrome (10). Females usually have normal gonadal functions. All 3 of our mal e patients also had testes small for their age, suggesting gonadal dysfunction. Unfortunately, we were not able to perform any hormonal investigations to evaluate gonadal function. 

Skeletal changes are frequently reported in 3M  syndrome. Slender long bones and ribs, foreshortened  lumbar vertebral bodies, tall vertebral bodies, short femoral neck, spina bifida occulta, small pelvis, small iliac wings and osteoporosis are common findings (1,2,3,10). Distal  shortening of the ulna and kyphoscoliosis are rarely reported in these patients (2). Our patients all showed the radiological features of the syndrome except for spina bifida occulta, shortening of the ulna and kyphoscoliosis. 

 The mode of inheritance is autosomal recessive in 3M  syndrome. CUL7 mutation in 6p21.1.  has been found in  several families (9,11). Recently, null mutations within the gene OBSL1 have also been reported (12). OBSL1 is a  putative cytoskeletal adaptor protein that localizes to the nuclear envelope OBSL1 and it has been proposed that its mutations are causing the primordial growth disorder 3M  syndrome (12). However, we were not able to perform genetic analyses in the patients presented in this report. 

 An early diagnosis is important for genetic counseling in 3M syndrome, especially in countries like Turkey where consanguineous marriages are common and autosomal  recessive genetic disorders leading to severe short stature should be kept in mind. 3M syndrome should always be  considered in the differential diagnosis of patients with growth retardation of prenatal onset. 
